# Editorial: Group dynamics and redistributive policy preferences in the Global South

**DOI:** 10.3389/fsoc.2023.1175466

**Published:** 2023-04-04

**Authors:** Liza G. Steele, Nate Breznau

**Affiliations:** ^1^Department of Sociology, John Jay College of Criminal Justice, New York, NY, United States; ^2^Department of Sociology, The Graduate Center, The City University of New York, New York, NY, United States; ^3^SOCIUM Research Center on Inequality and Social Policy, University of Bremen, Bremen, Germany

**Keywords:** preferences for redistribution, Global South, stratification, sociology, attitudes

## 1. Justification for Research Topic

Although social inequality is rising globally, increases in economic inequality and the unequal impact of poverty on diverse populations are most acute in the Global South. However, research on this topic is heavily biased toward the Global North where research suggests inequality reducing or social welfare enhancing policies are more successful where they have stronger public support (Nowlin, 2011; Jones and Baumgartner, 2012). Whether this is similar in the Global South has received less attention by scholars in part owing to less widely available polling and survey data (Sanjurjo, 2020). Although existing research points at some similar mechanisms in the richer Global South countries (Dorlach, 2021), it unequivocally focuses on structural and institutional explanations with almost no attention to public preferences (Yörük et al., 2022). Therefore, social science has far less to offer as a body of knowledge concerning the causes and consequences of attitudes toward inequality and preferences for redistributive social policies in the Global South.

In addition to developing knowledge about the Global South, this article collection aims to understand a wider trend of increasing global social, economic and political inequality. We seek to learn from the wider range of redistributive policies—or the expenditure of tax revenue on public goods and services—that is represented by all countries in particular outside of the 20 or so found in most studies. The wider range of countries is important for several reasons, including the fact that much social insurance legislation was pioneered by non-democratic regimes. Moreover, in the Global South, an overwhelming number of social insurance programs were initially adopted by non-democratic governments (Mares and Carnes, 2009).

We use a strict definition of the Global North, limiting that classification to the rich, colonizer empires of the world. For parsimony we define this as the high-income members of the Organisation for Economic Co-operation and Development (OECD), according to the World Bank's classification ^1^. All other countries are classified as being in the Global South, although authors in this special issue were free to adopt their own definitions.

## 2. Summary of selected literature

Using the *Social Policy Preferences Network* searchable bibliography^2^ ^2^, we review a selection of known comparative studies regarding redistributive policy preferences and policy outcomes in the Global South.

A study of elites in Brazil, South Africa and Uruguay (López et al., 2022) suggests they are strongly opposed to redistribution on average. This speaks directly to socio-economic position (“material self-interest”) as a strong factor in redistributive preferences, as found in studies of the Global North. Social structure as a determinant of redistributive preferences is borne out in a few studies of the Global South, or mixtures of the Global North and South countries. For example, former and satellite countries of the Soviet Union are highly supportive of redistribution on average, although this trend was strongest shortly after transition to market economies, suggesting that communist legacies likely have a long institutionalized impact on support for redistribution in the Global South (Okulicz-Kozaryn, 2014). In combined studies, preferences for redistribution (e.g., China and South Korea, Kitsnik, 2022) or general income egalitarianism (e.g., Poland and Bulgaria, Breznau, 2010) are structured by socio-economic status similar to Global North comparisons. An analogous comparative study of health insurance policy preferences points in a similar direction (Maldonado et al., 2019).

What is striking in studies combining Global North and South countries is that income inequality has a shaky association with redistributive preferences, if any at all (Steele, 2015; Breznau and Hommerich, 2019) and support for redistribution has little association with actual income transfers resulting from redistributive policy (Brady and Bostic, 2015); however, these findings may be biased due to the difficulty of measuring redistributive preferences in comparative surveys (Dallinger, 2022). In the East and Southeast Asian contexts this lack of association between inequality (and minimal income transfers) and support for redistribution may be a result of Asian values of self-determination and social duties (Chang, 2018).

## 3. Summary of Research Topic published articles

Attracting studies for this special issue was difficult for at least two reasons. The first is that the number of scholars who focus on the policy preferences in the Global South are far fewer than those in the Global North. The second is that survey data covering the Global South are scarce. Nonetheless, we received submissions that were diverse and innovative, that contribute on myriad fronts to the expansion of research in and on the Global South.

Representing universities in Chile and Spain, García-Sánchez et al., use data on Colombia to analyze one of the perennial questions in preferences for redistribution research: why preferences to reduce extreme inequalities often do not align with support of relevant policy options. The authors found that support for redistribution can be modeled as a latent construct depicting two different dimensions: one focused on taxing the wealthy and changing the income distribution schema, and other focused on assisting people in need and providing opportunities. The dimension related to taxing the wealthy (vs. assisting people in need) displayed higher internal reliability and correlated consistently with perceptions and attitudes toward inequality. Their research reviews distinct underlying dimensions of support for redistribution that shed light on different motivations that drive people's redistributive preferences.

Franetovic (Italy) and Castillo (Chile) re-examine the relationship between income and support for the reduction of inequalities through redistribution in Latin America, where research on the topic had seldom been conducted. Using data from the LAPOP Survey between 2008 and 2018, the authors are able to consider a longitudinal dimension is considered for the first time in the measurement of Latin American redistributive preferences. The results reveal that, unlike other regions, in Latin America it is not possible to detect a clear association between income and redistributive preferences at specific times, but it is possible when changes occur in countries' levels of inequality and economic development. Their findings challenge rationalist theories of justice and solidarity.

Immigration and welfare chauvinism—the idea that people support social spending more enthusiastically when the benefits go to people like themselves—are central concepts in the study of preferences for redistribution. The US-based team of Than et al. analyzed anti- and pro-immigrant attitudes regarding the 39 Vietnamese immigrants who died in a sealed lorry truck on their way to the UK (the “Essex Lorry Deaths”). Using machine learning methods (Structural Topic Modeling), they found that pro-immigration posts reflected counter-narratives that challenged the mainstream media's coverage of the incident and critiqued the militarization of borders and the criminalization of immigration. Anti-immigration posts ranged from reproducing stereotypes about Vietnamese immigrants to explicitly blaming the victims themselves or their families for the deaths. These events occurred in the shadow of Brexit, a political move closely associated with welfare chauvinism.

## Author contributions

Both authors listed have made a substantial, direct, and intellectual contribution to the work and approved it for publication.

## Footnotes

^1^ http://data.worldbank.org/about/country-classifications/country-and-lending-groups#OECD_members

^2^https://sites.google.com/view/sppn/bibliography (based on the bibfilter app https://github.com/Tomptez/Bibfilter).
